# Pre-transplant infusion of donor leukocytes treated with extracorporeal photochemotherapy induces immune hypo-responsiveness and long-term allograft survival in murine models

**DOI:** 10.1038/s41598-022-11290-w

**Published:** 2022-05-04

**Authors:** Jennifer Schneiderman, Longhui Qiu, Xin Yi Yeap, Xin Kang, Feibo Zheng, Junsheng Ye, Yan Xie, Jiao-Jing Wang, Yuvaraj Sambandam, James Mathew, Lin Li, Joseph Leventhal, Richard L. Edelson, Zheng Jenny Zhang

**Affiliations:** 1 Department of Pediatrics, Hematology/Oncology/Neuro-Oncology/Stem Cell Transplantation and Cellular Therapy Program, Feinberg School of Medicine, Ann and Robert H. Lurie Children’s Hospital of Chicago, Northwestern University, Chicago, IL USA; 2 Department of Surgery, Comprehensive Transplant Center, Feinberg School of Medicine, Microsurgery Core, Northwestern University, Chicago, IL USA; 3 Department of Pathology and Laboratory Medicine, Cedars Sinai Medical Center, West Hollywood, CA USA; 4 Department of Surgery, Organ Transplantation, Feinberg School of Medicine, Kidney and Pancreas Transplant Programs, Northwestern University, Chicago, IL USA; 5 Department of Dermatology, Yale School of Medicine, New Haven, CT USA

**Keywords:** Immunology, Antigen processing and presentation, Transplant immunology, Preclinical research

## Abstract

Recipients of solid organ transplantation (SOT) rely on life-long immunosuppression (IS), which is associated with significant side effects. Extracorporeal photochemotherapy (ECP) is a safe, existing cellular therapy used to treat transplant rejection by modulating the recipient’s own blood cells. We sought to induce donor-specific hypo-responsiveness of SOT recipients by infusing ECP-treated donor leukocytes prior to transplant. To this end, we utilized major histocompatibility complex mismatched rodent models of allogeneic cardiac, liver, and kidney transplantation to test this novel strategy. Leukocytes isolated from donor-matched spleens for ECP treatment (ECP-DL) were infused into transplant recipients seven days prior to SOT. Pre-transplant infusion of ECP-DL without additional IS was associated with prolonged graft survival in all models. This innovative approach promoted the production of tolerogenic dendritic cells and regulatory T-cells with subsequent inhibition of T-cell priming and differentiation, along with a significant reduction of donor-specific T-cells in the spleen and grafts of treated animals. This new application of donor-type ECP-treated leukocytes provides insight into the mechanisms behind ECP-induced immunoregulation and holds significant promise in the prevention of graft rejection and reduction in need of global immune suppressive therapy in patients following SOT.

## Introduction

Solid organ transplant (SOT) recipients are typically committed to life-long treatment with immunosuppressive therapies (IS) to prevent graft rejection. However, despite significant advances in the field, patients remain at risk for acute and chronic rejection. Chronic use of IS increases the risk of infection, secondary malignancies, cardiovascular disease, dyslipidemia, and diabetes^1,2^. These challenges highlight the importance of establishing novel approaches that minimize or eliminate the need for prolonged IS through the induction of donor-specific hypo-responsiveness of the host’s immune system to the graft.

As such, cellular therapy has moved to the forefront of small animal and clinical research. Co-transplantation of allogeneic hematopoietic stem cells (HSC) and a solid organ, and infusion of either manipulated host or donor regulatory cells are being studied^3–8^. While promising, HSC co-transplantation comes with significant risks associated with the preparative regimen, and potential for graft-versus-host disease and associated profound immunosuppression^9–11^. Utilizing infusion of modified autologous dendritic cells (DC) or regulatory T-cells (Tregs) are limited by expense of manipulation, relatively small numbers of cells obtained and have yet to realize promise in clinical application as treated subjects often ultimately require re-initiation of IS. The infusion of donor immunoregulatory cells has been investigated in pre-clinical rodent models, with successful demonstration of tolerance induction^12,13^. Phase I studies of manipulated donor cell infusions in humans have been safe, without infusional toxicities or evidence of sensitization^6,11^. Most studies to date have utilized one-time cell infusions; effects may not have enough clinical longevity to avoid additional IS therapy for the life of the graft and patient.

Extracorporeal photochemotherapy (ECP) is a readily available, safe apheresis therapy used to treat rejection following SOT. Early studies suggested ECP may have a positive effect in the prevention of rejection^14–16^; the current clinical application of ECP is as second- or higher line therapy in organ rejection. ECP involves the collection of *autologous* peripheral blood mononuclear cells (PBMCs), to which 8-methoxypsoralen (8-MOP) is added; cells are exposed to UV-A light and returned to the patient. It has a well-established safety record over decades and does not induce global immune suppression^17–19^. Reports have been published in recipients of heart, lung, liver, kidney, and composite tissue transplants^20–26^. Current clinical practice is limited to treatment of *autologous recipient* cells. Given the promise of utilizing modified donor-derived cell infusions for tolerance induction, we repurposed ECP in a novel way: utilizing *donor-derived* cells (ECP-DL) pre-transplant in MHC-disparate murine heterotopic cardiac, and orthotopic liver and kidney transplantation models.

We hypothesized that administering ECP-DL pre-transplant would down-regulate host immune responses in an antigen-specific manner, facilitating induction of donor-specific hypo-responsiveness, and that combining ECP-DL infusions with conventional post-transplant recipient ECP treatment (ECP-RL) would provide a protective umbrella under which a significant tolerogenic effect would be maintained long-term. Herein, we demonstrate that a single infusion of ECP-DL significantly prolongs cardiac, kidney and liver allograft survival in a donor-specific manner across MHC barriers; addition of short-course IS provides synergistic prolongation of graft function. We present initial insight into the underlying cellular effects potentially responsible for the hypo-responsiveness observed. This is the first report demonstrating the presence of ECP-induced tolerogenic myeloid cells capable of local reduction of inflammation and promotion of tissue repair. Infusion of ECP-DL prior to SOT followed by ongoing ECP-RL treatment post-transplant may serve as an effective strategy for induction and subsequent maintenance of donor-specific hypo-responsiveness, permitting long-term graft acceptance without the need for excessive IS therapy.

## Results

### A single infusion of ECP-DL significantly prolonged survival of cardiac allografts via downregulation of T-cell activation, favoring Treg production

**Figure 1:**
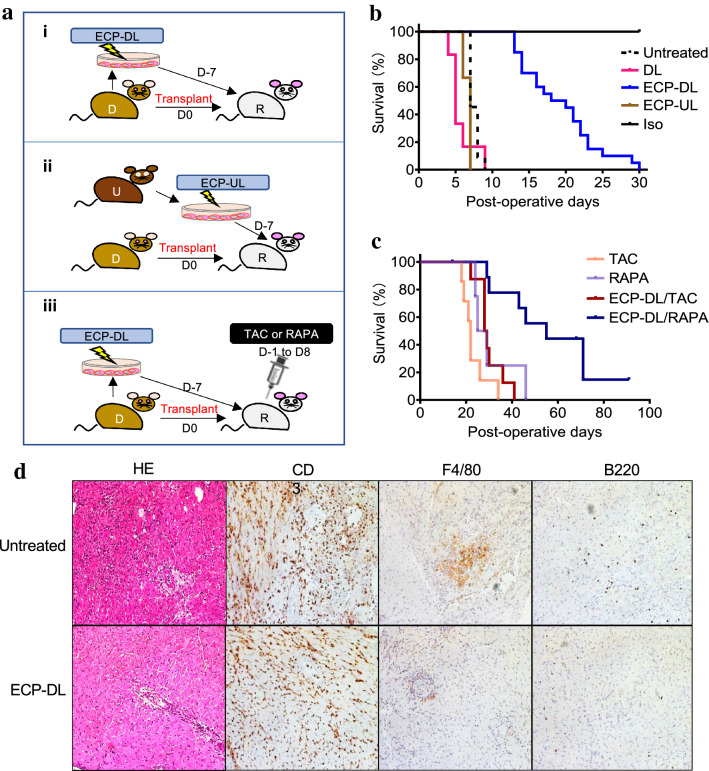
ECP-DL infusion prior to transplantation significantly prolonged cardiac allograft survival in MHC-mismatched mouse models; the addition of TAC or RAPA was synergistic and resulted in further improvement of graft survival. Mouse models of heterotopic cardiac transplantation (BALB/c to B6) were used to test the efficacy of ECP-DL in cardiac allograft rejection. (**a**) Pictorial representation of models utilized. ECP-DL from BALB/c into B6 on D-7 followed by heart transplant on D0 (i); ECP-UL from third party (C3H) into B6 on D-7 and heart from BALB/c on D0 (ii); ECP-DL on D-7 and heart on D0 from BALB/c into B6 plus short-course TAC or RAPA from D-1 to D8 (iii). (**b**) Kaplan Meier Curves demonstrating that ECP-DL infusion (n = 20) resulted in prolonged cardiac allograft survival compared to Untreated (n = 11), DL (n = 6), and ECP-UL (n = 3) groups. The Isograft group survived indefinitely (n = 6). ECP-DL versus Untreated, DL, or ECP-UL, ^****^p < 0.0001, by Log-rank test. (**c**) Kaplan Meier Curves showing ECP-DL was synergistic with TAC or RAPA. TAC (n = 8); RAPA (n = 4); ECP-DL/TAC (n = 8); ECP-DL/RAPA (n = 10). ECP-DL/RAPA versus RAPA, ^*^p < 0.05; ECP-DL/TAC versus TAC, ^*^p < 0.05; by Log-rank test. (**d**) Representative hematoxylin and eosin, CD3, F4/80, and B220 staining of cardiac allografts from untreated versus mice receiving ECP-DL. Grafts from ECP-DL treated animals had reduced inflammatory cellular infiltrates. The composition of the infiltrates was further characterized by the staining of CD3 (T-cells), F4/80 (monocyte/macrophages), and B220 (B-cells).

We evaluated the effect of infusing ECP-DL in a heterotopic mouse heart transplant model (27); treatment groups utilized are illustrated in Fig. 1ai-iii. B6 mice were recipients, and BALB/c mice were donors of ECP-DL on day -7 and heart on day 0 (Fig. 1ai). In the absence of IS, this donor-recipient combination results in loss of palpable heart impulse in approximately 10 days. ECP-treated splenocytes from MHC-mismatched third-party mice (C3H) were administered to B6 mice on day -7 before transplant from BALB/c donors in a subset of experiments to test donor specificity (Fig. 1aii). To test the effect of IS, tacrolimus (TAC) or rapamycin (RAPA) was added from day -1 to post-operative day (POD)8 to ECP-DL treatment (Fig. 1aiii). Syngeneic heart transplants served as controls (Iso, Fig. 1b). Pre-transplant infusion of ECP-DL was effective in prolonging allograft survival; animals receiving ECP-DL alone had significantly prolonged graft survival until POD30, whereas allografts receiving no therapy (Untreated), unmanipulated donor-type splenocytes (DL), or ECP-treated third-party cells (ECP-UL) all lost palpable impulse of the graft by POD10 (Fig. 1b). Combined with TAC or RAPA, further prolongation of allograft survival was observed; the combination of ECP-DL and RAPA demonstrated the longest duration of cardiac impulse (Fig. 1c). We also tested administration of ECP-DL on days -3, and -1 and found no statistical difference in graft survival (Supplemental fig 1a). Finally, experiments were performed utilizing a reversal of donor and recipient strains (B6 grafts/ECP-DL into BALB/c mice) to correct for known differences in strain combination; results were identical to those reported above (Supplemental fig 1b). A subset of animals receiving no treatment and those receiving ECP-DL were sacrificed on POD7 to examine the grafts histologically. Grafts from untreated animals had increased inflammatory infiltrate compared to those from ECP-DL animals. We performed stains to identify T-cells (CD3), monocyte/macrophages (F4/80), and B-cells (B220); untreated grafts had more prominent infiltration of all inflammatory cell types (Fig. 1d).

**Figure 2:**
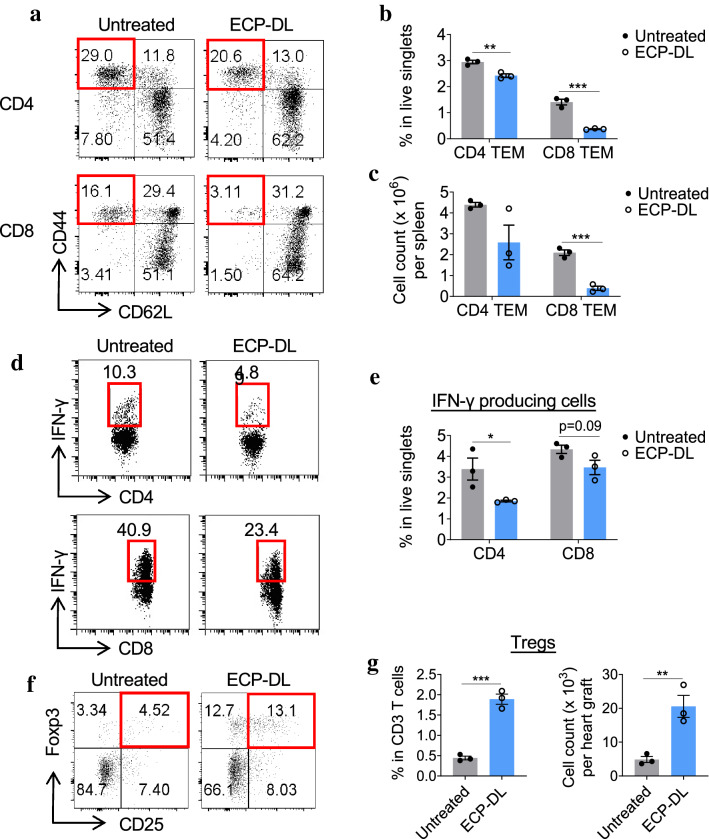
ECP-DL infusion inhibits effector memory T-cells and Th1 response after cardiac transplantation. ECP-DL treated or untreated controls were euthanized on POD7; spleens and heart grafts were harvested for flow cytometry analysis (n = 3 per group). (**a-c**) Representative dot plots and bar graphs showing that in ECP-treated mice, both CD4^+^ and CD8^+^ effector memory T-cells (TEM, CD44^+^CD62L^-^) were decreased compared with that of the untreated group. (**d, e**) The frequency of interferon-gamma-producing CD4^+^ and CD8^+^ T-cells was significantly decreased in the ECP-treated mice, demonstrating a reduced Th1 response. (**f, g**) Tregs, identified as CD25^+^/Foxp3^+^ (gated from CD3^+^CD4^+^ cells) were significantly higher in the cardiac allografts of the ECP-DL group than that of the untreated group. In **B**, **C**, **E**, and **G**, ^*^p < 0.05, ^**^p < 0.01, ^***^p < 0.001 by student *t*-test.

To evaluate the effect of ECP-DL infusion on T-cell response, transplant recipients receiving either ECP-DL on day -7 or no treatment were sacrificed on POD7. Lymphocytes from the spleen were recovered and subjected to flow cytometry; activation of CD4^+^ and CD8^+^ T-cells in the ECP-treated mice was significantly reduced as indicated by decreased CD44^+^CD62L^-^ effector T-cell populations (Fig. 2a–c). Secretion of IFN-γ from both CD4^+^ and CD8^+^ T-cells was lower in the ECP treated group, indicating a reduced inflammatory response (Fig. 2d, e); this was statistically significant in the CD4^+^ T-cell fraction. The frequency of Tregs was significantly increased in treated mice (13% vs 4.5% in untreated animals, Fig. 2f, g). Together, these data illustrate that in an antigen-specific manner, ECP-DL treatment prolongs graft survival, decreases graft infiltrating inflammatory cells, down-regulates T-cell activation, and promotes Treg production.

### A single infusion of ECP-DL decreased production of antigen-specific CD4^+^ and CD8^+^ T-cells and suppressed their proliferation through direct antigen presentation

**Figure 3:**
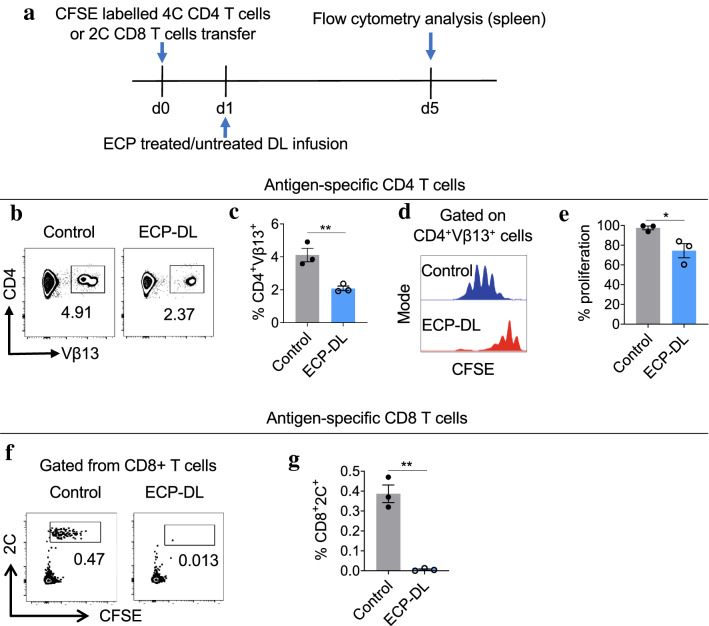
ECP-DL infusion decreases antigen-specific CD4^+^ and CD8^+^ T-cells and suppresses their proliferation prior to cardiac transplantation. (**a**) Transgenic B6 mice (4C and 2C) were utilized to evaluate the effect of ECP-DL treatment on alloreactive T-cells. CD4-4C and CD8-2C cells were isolated from transgenic B6 mice and labeled with CFSE; cells were then injected into B6 mice (d0). On day 1, ECP-DL or untreated splenocytes from BALB/c mice were infused. On day 5, recipient mice were sacrificed, and antigen-specific T-cells in the spleen were assessed. N = 3 per group. (**b, c**) Representative dot plots and bar graphs demonstrating antigen-specific CD4^+^ T-cells (Vβ13^+^CD4^+^, gated from CD3^+^CD4^+^ T-cells) were significantly lower in the spleen of mice treated with ECP-DL compared to that of the untreated group. (**d, e**) The proliferation of antigen-specific CD4^+^ T-cells isolated from the spleen was significantly lower in the ECP-DL treated group as measured by the CFSE intensity. (**f, g**) Representative dot plots and bar graphs demonstrating antigen-specific CD8^+^ T-cells (2C^+^CFSE^+^, gated from CD3^+^CD8^+^ T-cells) were significantly lower in the spleen of mice treated with ECP-DL compared to that of the untreated group. Their proliferation rate was not compared as the frequency of 2C^+^ CD8 T-cells in the ECP-DL group was almost undetectable. In **c**, **e**, and **g**, ^*^p < 0.05, ^**^p < 0.01 by student *t*-test.

Subsequently, we utilized an established 2C and 4C transgenic mouse model to evaluate the antigen-specific effects of ECP therapy on alloreactive T-cells prior to transplantation^27^. CD-4C and CD8-2C T-cells were isolated from TCR transgenic B6 mice and labeled with carboxyfluorescein succinimidyl ester (CFSE) to evaluate lymphocyte proliferation rates. CD4-4C and CD8-2C T-cells recognize epitopes presented by donor MHC class II or I molecules, respectively. Labeled T-cells from transgenic B6 mice were injected into standard B6 mice on day 0. On day 1, splenocytes from standard BALB/c mice were isolated as in prior experiments and were injected into the B6 recipient; splenocytes were either ECP-treated or not. On day 3 B6 animals were sacrificed to assess the frequency of T-cells in the spleen; none underwent transplant. These procedures are depicted in Fig. 3a.

Treatment with ECP resulted in a statistically significant depletion in the number of antigen-specific CD4^+^ T-cells found infiltrating the spleen compared to untreated animals, by 48% (Fig. 3b, c). CFSE concentration within the antigen-specific CD4^+^ T-cells in ECP-treated animals was higher, indicating reduced proliferation; this was also statistically significant (Fig. 3d, e). Results in the CD8^+^ compartment were striking: proliferation within treated animals was essentially absent compared to high levels of proliferation seen in control animals (Fig. 3f, g).

Given the remarkable difference between groups in the CD8 compartment, an additional set of experiments were performed to evaluate the fate of CD8 cells after transplant; B6 mice were injected with CFSE-labeled CD8-2C cells on day -8. On day -6, the animals were given either ECP-treated or untreated splenocytes; heart transplant was performed on day 0 and animals were sacrificed on POD5. Flow cytometry was performed on cells isolated from the spleen and graft (Supplemental fig 2a). There were substantially fewer infiltrating CD8^+^ cells in treated animals (Supplemental fig 2b). Collectively, the 2C/4C transgenic mouse experiments illustrate a drastic reduction of antigen-specific alloreactive T-cells in ECP-treated animals, which, when present, are less proliferative.

### A single infusion of ECP-DL inhibited production of donor specific antibodies (DSA)

**Figure 4:**
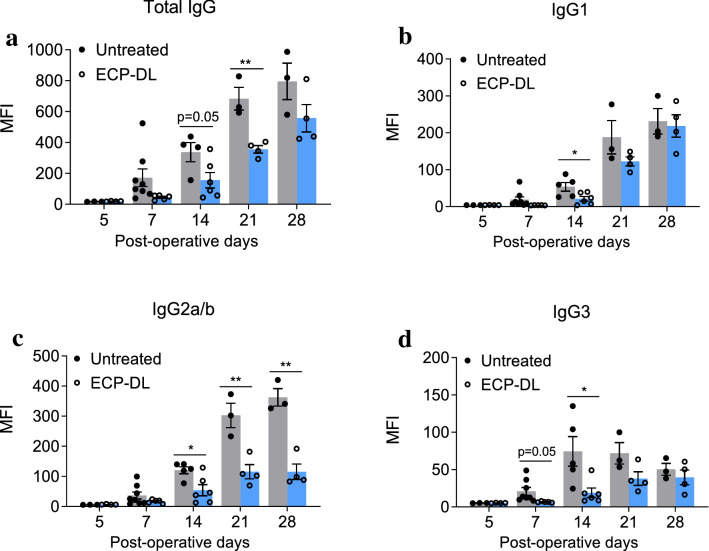
Treatment with ECP-DL inhibits the production of donor-specific antibodies (DSA) following heterotopic heart transplants. After heart transplantation (BALB/c to B6), plasma samples were collected from B6 recipient mice and assessed for DSA at POD5, 7, 14, 21, and 28 (n = 3–8 for each timepoint). Transplant recipients either received ECP-DL at day -7 or no treatment. Production of all DSA subtypes was lower in ECP-DL treated animals at all time points compared to control. Minimal DSA production in the ECP-DL group at POD5 suggests that the animals are not sensitized by ECP-DL infusion. MFI: Mean fluorescence intensity; ^*^p < 0.05, ^**^p < 0.01 by two-way ANOVA, followed by classical t-tests.

We next asked how ECP-DL infusions affected B-cells by evaluating production of DSA. We performed cardiac transplants in two groups of animals, untreated and those receiving ECP-DL on day -7. DSA were measured at POD5, 7, 14, 21, and 28. Total IgG (Fig. 4a), and subtypes IgG1 (Fig. 4b), IgG2 a/b (Fig. 4c), and IgG3 (Fig. 4d) were assessed. Importantly, minimal DSA were detected in the ECP-DL-treated animals at POD5, suggesting they are not sensitized following ECP-DL treatment. At all timepoints, DSA were lower in the treated group; the differences were statistically significant across all antibody subtypes at most times. This data was reproduced in our liver transplant model (data not shown).

### ECP induced apoptosis; most apoptotic cells were phagocytosed in the spleen

We sought to determine the fate of ECP-DL in non-transplanted animals. BALB/c splenocytes were treated with ECP and subjected to Annexin V staining. Approximately half of ECP-DL became apoptotic prior to infusion (Supplemental Fig 3a). We subsequently performed experiments in which ECP-DL were labeled with PKH26 dye and injected into B6 mice for tracking (without transplant). Fifteen hours after injection, mice were sacrificed and spleens analyzed; the intensity of PHK26 was tested in monocytes (CD11b^+^/Ly6C^+^), granulocytes (CD11b^+^/Ly6G^+^), and macrophages (CD11b^+^/F4/80^+^). Around 6% of CD45^+^ leukocytes and up to 66% of macrophages (CD11b^+^F4/80^+^) took up PKH dye (Supplemental Fig 3b). Within the myeloid line, PKH26 signal was detectible primarily in macrophages but not in neutrophils or monocytes, indicating that ECP-DL which had undergone apoptosis had been phagocytosed by macrophages in the spleen (Supplemental Fig 3c). Together, these data indicate that ECP-DL, once injected, become apoptotic and are taken up by recipient macrophages in the spleen, allowing for donor antigen processing by myeloid cells.

### Infusion of ECP-DL led to localization of DCs with tolerogenic phenotype in the allografts

**Figure 5:**
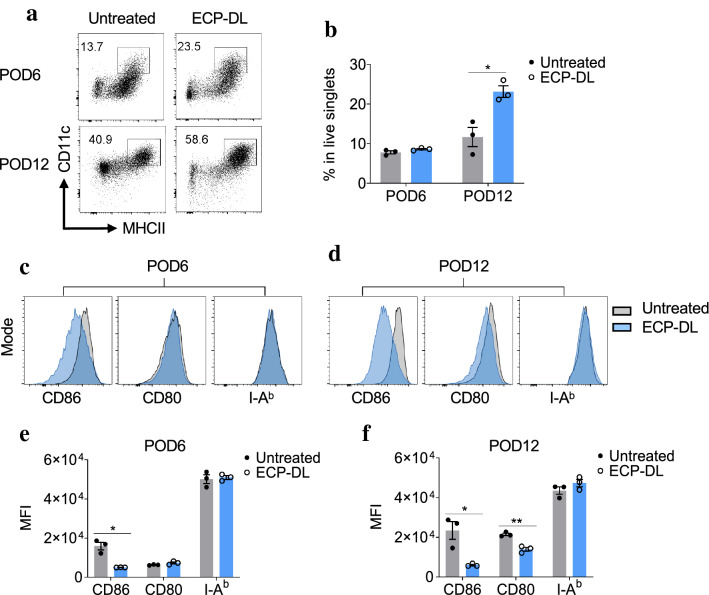
ECP-DL infusion increased the percentage of tolerogenic host dendritic cell infiltrates in heart allografts. Both groups underwent heterotopic heart transplants and either received ECP-DL on day -7 or no therapy; animals were sacrificed on POD6 or 12; heart grafts infiltrating cells were isolated and subjected to flow cytometry analysis (n = 3/group). Cells were gated on H-2b^+^ (host) CD11b^+^ live single cells. (**a, b**) Representative dot-plots and bar graphs showing the percentage of host-derived dendritic cells infiltrating in the graft at POD6 and POD12. ^*^p < 0.05 by student *t*-test. (**c, d**) Representative histogram and (**e, f**) corresponding bar graphs of CD86, CD80, and MHCII (I-A^b^) expression of graft-infiltrating dendritic cells. Host-derived dendritic cells isolated from the grafts in ECP-DL treated mice express significantly lower levels of CD86 at both POD6 and POD12. MFI: Mean fluorescence intensity; ^*^p < 0.05, ^**^p < 0.01 by student *t*-test.

Given the known regulatory effect of DC on the state of T-cell activation and transplant tolerance, we sought to characterize graft infiltrating DC. Heterotopic heart transplants were performed with or without a single infusion of ECP-DL on day -7, and we evaluated the number and phenotype of infiltrating cells within the graft on POD6 and POD12. Gating strategies utilized to evaluate infiltrating DC are provided in Supplemental Fig. 4. Host DC population was identified within the H-2Db/CD11b gate and is MHC II^+^/CD11c^+^. At POD6 there was a trend to increased DC within the grafts retrieved from treated animals compared to control, but this difference was not statistically significant. At POD12, however, statistical significance was achieved (Fig. 5a, b). To better characterize the functional phenotype of these cells, we evaluated the change in co-stimulatory molecule expression CD86, CD80, and MHC Class II (I-A^b^, Fig. 5c–f). Interestingly, CD86 expression on dendritic cells is statistically significantly reduced at both POD6 and POD12 in the grafts of ECP treated animals. CD80 expression on graft infiltrating DC were increased at POD6 but then reduced compared to controls at POD12. Additionally, MHC class II expression is higher on the graft infiltrating DC from ECP-treated animals. Taken together, we demonstrate an increase in DC infiltrating the grafts of ECP-treated mice, which exhibit a tolerogenic phenotype.

### ECP-DL treatment led to an increase in anti-inflammatory and pro-reparative macrophages infiltrating the graft

To determine whether ECP-DL influences the recruitment of myeloid cells into the allograft, we enumerated and characterized host myeloid cells (e.g. monocyte/macrophages, granulocytes) in the allografts on POD6 and POD12 by using a Ly6C/Ly6G-based strategy (Supplemental Fig 5a–c)^28^. We found that infiltrating host (H-2D^b+^) myeloid cells primarily consist of activated CD11b^+^ Ly6G^+^/Ly6C^int^ cells (granulocytes) and CD11b^+^Ly6G^-^/Ly6C^hi^ cells (macrophages) at POD6 and POD12 based on their expression of costimulatory molecules CD80, CD86 and MHC class II (Supplemental fig 5d–g). ECP-DL infusion significantly reduced the number of granulocytes in grafts at POD6 and POD12 compared to untreated controls. Interestingly, the number of macrophages infiltrating the grafts of treated animals were reduced compared to control at POD6, while at POD12 there were more; the differences at both time points were statistically significant. CD86 expression on infiltrating macrophages (Supplemental Fig 5h–k) was significantly downregulated in the ECP treated animals compared to controls. In this cell population, CD80 expression was decreased compared to control animals at both time points, while MHC Class II expression was lower in ECP treated animals at POD6 but higher than controls at POD12. Together, these data indicate ECP-DL treatment not only reduced number of graft infiltrating CD11b^+^ Ly6G + /Ly6C^int^ cells and CD11b^+^Ly6G^-^/Ly6C^hi^ myeloid cells, but also regulated differentiation of these myeloid cells by down-regulating expression of co-stimulating molecules, particularly on CD11b^+^Ly6G^-^/Ly6C^hi^ cells, suggesting that the cells present tended towards an anti-inflammatory phenotype over time.

**Figure 6:**
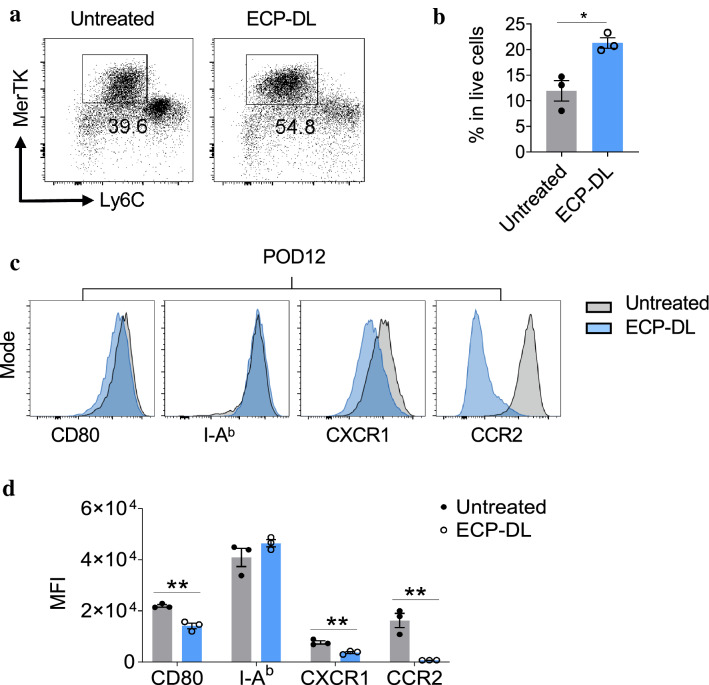
ECP-DL infusion led to an increased accumulation of MerTK-expressing macrophages in allografts. BALB/c to B6 cardiac transplants were performed; animals received ECP-DL on day -7 or no therapy and were sacrificed on POD12. Grafts were harvested and infiltrating cells were subjected to flow cytometry. (**a, b**) Representative flow cytometric plots (gated from H2Db^+^CD11b^+^ live single cells) with corresponding bar graphs demonstrating higher percentage of MerTK^+^ macrophages in the ECP-DL compared to the untreated group (n = 3/group). *p < 0.05 by student *t*-test. (**c, d**) Representative histogram (gated from Ly6C^low^MerTK^+^ population) and corresponding bar graphs, demonstrating the graft-infiltrating MerTK^+^ macrophages had a significant phenotypic difference between the ECP-DL and untreated group. Expression levels of CD80, CXCR1, and CCR2 in MerTK^+^ cells from the ECP-DL group were all significantly lower than those from the untreated group, indicating ECP-DL promotes anti-inflammatory and pro-reparative infiltrates in the allografts. MFI: Mean fluorescence intensity; ^**^p < 0.01 by student *t*-test.

To further define the monocyte population infiltrating the grafts at POD12, we evaluated the number and phenotype of monocytes expressing MerTK. There was a significantly higher number of MerTK expressing monocytes in the ECP-DL treatment group compared with control; most cells that were MerTK positive in the ECP treated grafts were also Ly6C negative, indicating a less inflammatory phenotype (Fig. 6a, b). Importantly, these monocytes were also phenotypically different in ECP-DL treated vs untreated animals, in that expression of CD80, CCR2, and CXCR1 expression were all significantly lower in the grafts from ECP-DL animals. MHC class II expression was higher in the ECP treated group (Fig. 6c, d). The significant increase in MerTK expression along with the reduction of Ly6C expression indicates the presence of tolerogenic monocytes capable of promoting a favorable cytokine milieu, promote Treg production, negatively regulate nuclear factor kappa B (NF-κb) expression, and promote tissue repair through efferocytosis.

### A single infusion of ECP-DL significantly prolonged the survival of liver and kidney allografts, maintained long-term allograft function and histologic appearance

We subsequently looked to confirm these promising results in orthotopic kidney and liver transplants in rats, which are functional transplants in that entire native kidneys and liver in each model are removed; they are also more immunologically robust than the cardiac model. We first evaluated a rat orthotopic kidney transplant model; Lewis rats were recipients, and MHC-mismatched ACI rats were donors. Terminal rejection will develop in less than two weeks without IS in this high-responder pair^29,30^. As expected, untreated allogeneic recipients died of renal failure in the first 10 days after transplantation (Untreated, Supplemental Fig. 6a). In stark contrast, ECP-DL pre-conditioning on day -7 significantly prolonged allograft survival to more than 50 days. Moreover, 60% of animals receiving ECP-DL alone survived to day + 200. While less than 40% of those receiving TAC alone survive, all animals receiving combination ECP-DL plus TAC survived indefinitely (Supplemental Fig. 6a), demonstrating robust synergy. Creatinine levels were monitored in the blood at pre-determined times. Animals receiving either ECP-DL, TAC, or combination therapy had normal levels of creatinine at POD7 and POD90 (Supplemental Fig. 6b). Grafts were examined histologically at the time of sacrifice (time of renal failure or POD200). Kidneys from rats receiving ECP-DL appear like isografts without significant damage to glomeruli or collecting ducts, while those from untreated animals demonstrated damage consistent with rejection (Supplemental fig 6c). Together, these results illustrate that even in a highly immunogenic kidney transplant model, ECP-DL treatment leads to improved graft survival; addition of short-course IS was synergistic and led to indefinite survival.

**Figure 7:**
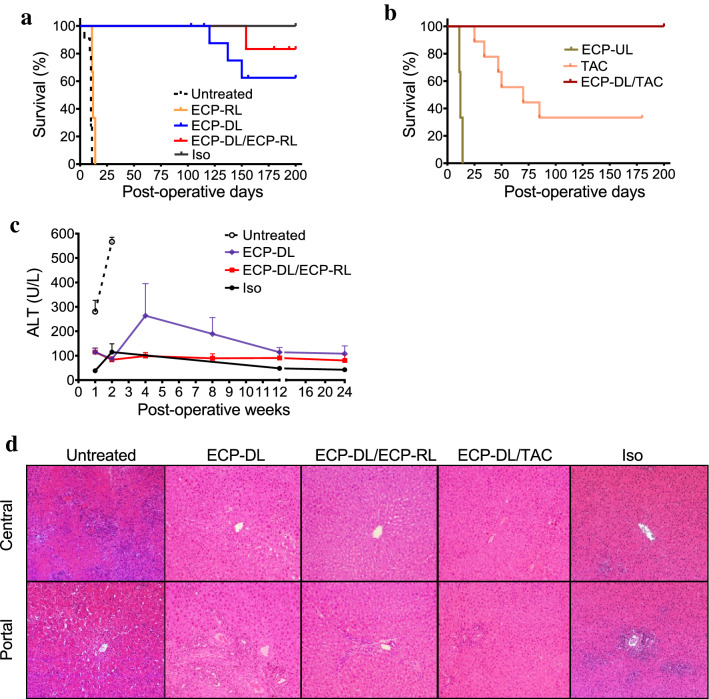
A single infusion of ECP-DL significantly prolonged the survival of liver allografts, while maintaining normal liver function and histologic appearance. Liver transplants from ACI rats into Lewis rats. (**a** and **b**) Kaplan Meier survival curves showing ECP-DL significantly prolonged the survival of liver transplant recipients and was synergistic with ECP-RL or a short course treatment with TAC. Untreated, n = 11; ECP-RL, n = 3; ECP-DL, n = 10; ECP-DL/ECP-RL, n = 6; Iso, n = 3; ECP-UL, n = 3; TAC, n = 9; ECP-DL/TAC, n = 4. ECP-DL versus Untreated or ECP-RL, ^****^P < 0.0001; ECP-DL/TAC versus TAC, ^*^P < 0.05, by Log-rank test. (**c**) ALT testing revealed overall preservation of liver function in ECP-DL and ECP-DL/RL recipients despite an initial rise at week 4 in the ECP-DL group. Mean ± SEM; n = 3–6 for each timepoint. Untreated versus ECP-DL or ECP-DL/ECP-RL, ^**^P < 0.0001 at the first post-operative week, ^****^P < 0.0001 at the second post-operative week by the unpaired *t*-test. (**d**) Hematoxylin and eosin stains of liver grafts at the time of sacrifice reveal that liver tissue from untreated animals have a significant amount of infiltrative inflammatory cells; liver tissue retrieved from ECP-DL and ECP-DL/ECP-RL animals had near-normal histology like isograft controls.

Similar experiments utilizing ACI rats as cell and liver donors and Lewis rats as recipients were then performed. Syngeneic liver transplants (Lewis/Lewis) served as controls (Iso); as expected, they survived indefinitely (> 200 days). Both untreated allograft recipients and allografts receiving ECP-RL (ECP-treated autologous splenocytes) infusion pre-transplant experienced rejection and died within two weeks. Strikingly, 60% of allogeneic animals receiving a single infusion of ECP-DL seven days prior to SOT survived to POD200 without additional therapy; this difference was statistically significant. Adding post-transplant autologous ECP-RL infusions at POD14 and POD28 to ECP-DL pre-conditioning on day -7 further promoted allograft survival, with 80% of ECP-DL/ECP-RL animals surviving to POD200 (Fig. 7a); this improvement in survival was statistically significant. Figure 7b shows the survival of animals that were treated with TAC + /− ECP-DL infusion. TAC alone prolonged allograft survival with 38% of animals surviving to study endpoint, while allografts receiving pre-transplant ECP-DL infusion plus TAC survived indefinitely, demonstrating a marked synergistic effect. Notably, recipients of ECP-treated unrelated third-party cells (ECP-UL) prior to transplantation were not protected, again illustrating a donor-specific effect (Fig. 7b). Serum was tested at specified intervals to evaluate liver function. Despite an initial rise in alanine aminotransferase (ALT) in the ECP-DL group at week 4, levels approached those of the ECP-DL/ECP-RL and isograft groups by week 24 (Fig. 7c). Histological analysis of the transplanted liver revealed that allografts in the ECP-DL, ECP-DL/RL, and ECP-DL/TAC groups maintained intact architecture and had minimal cellular infiltrates, comparable to isografts, while animals receiving no therapy had disruption of portal triads and cellular infiltration consistent with rejection (Fig. 7d).

**Figure 8:**
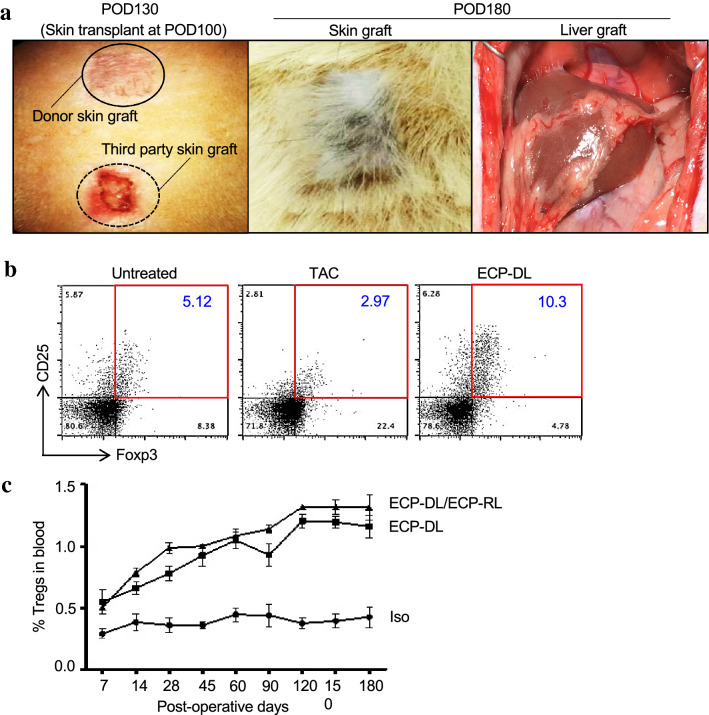
ECP-DL induced hypo-responsiveness is donor-specific and is associated with increased frequency of circulating T-regulatory cells. Lewis rats received orthotopic liver grafts from ACI donors 7 days after injection of ECP-DL without additional IS. Skin grafts from both donor (ACI) and third party (BN) rats were applied to the same recipient 100 days post-liver transplant to determine whether the tolerance is donor specific. Animals were monitored, and serial blood samples were analyzed by flow cytometry for the frequency of Tregs. (**a**) Representative gross images showing the donor-type skin grafts at post-operative (POD) 130 versus third-party skin grafts. By POD 180, the donor-type skin grafts further improved in appearance, while third-party skin grafts ulcerated, indicating rejection; the liver graft appears normal in situ. (**b**) Representative dot-plot showing % of Tregs, and a bar graph of the number of Tregs in the blood at POD7 showing higher percentage and absolute numbers of Tregs in ECP-DL treated recipients than the untreated or those receiving short-course tacrolimus (TAC). (**c**) The percentage of Tregs over the course of the experiment; animals receiving either ECP-DL or ECP-DL/ECP-RL had significantly higher percentages of Tregs than isograft controls at all time points.

Finally, to further confirm that immune hypo-responsiveness is donor-specific, additional animals received ECP-DL seven days prior to liver transplant, and subsequently underwent skin transplant from either ACI or a third-party rat (Brown Norway, BN) at day + 100. By day + 130 the skin graft from BN had ulcerated (indicating rejection), while donor-derived skin allografts healed well, progressing to a normal appearance by time of sacrifice at day + 180 (Fig. 8a). The successful engraftment of donor-origin skin grafts contrasted with rejection of third-party grafts provides further evidence that the effects of ECP-DL pre-conditioning are donor specific. The transplanted liver in animals receiving a single infusion of ECP-DL maintained normal gross appearance at the time of sacrifice (Fig. 8a).

We hypothesized that CD4^+^/CD25^+^/FoxP3^+^Tregs were involved in the hypo-responsiveness induced by ECP-DL; flow cytometry was performed on peripheral blood at specified intervals post-transplant. When comparing untreated animals to those receiving TAC and day -7 ECP-DL (on POD7), the ECP-DL group had a higher number of Tregs (Fig. 8b). Longitudinally, both the ECP-DL and ECP-DL/ECP-RL groups had similar and significantly higher number of Tregs in the peripheral blood at all time points compared to the isograft group (Fig. 8c). Collectively, these experiments reinforce that this is an antigen specific process, and that treated animals have significantly higher levels of circulating Tregs compared to control over time.

Overall, our results have shown that while pre-conditioning with a single infusion of ECP-DL resulted in long-term allograft survival, infusion with ECP-treated *autologous* cells prior to transplant did not protect the graft, demonstrating that the effect seen in the ECP-DL group was not simply because of the infusion of apoptotic cells. Furthermore, pre-transplant infusion of ECP-DL plus post-transplant infusions of ECP-RL or TAC were synergistic in facilitating immune hypo-responsiveness to both liver and kidney allografts promoting indefinite survival.

## Discussion

Herein we present an innovative and easily repurposed approach to ECP, which was successful in abating rejection in multiple experimental models across MHC barriers. To our knowledge, these experiments are the first to demonstrate that administration of donor-type leukocytes treated with ECP prior to SOT induces antigen-specific immune hypo-responsiveness. Furthermore, these are the first experiments demonstrating that *donor* cells treated with ECP induce the reparative pathway of efferocytosis in addition to the stimulation of Tregs, tolerogenic monocytes and macrophages, reduce trafficking and activation of inflammatory cells to the graft, and delay donor-specific antibody production, collectively protecting graft function. Importantly, the effect of ECP-DL induction therapy was confirmed in multiple organ transplant models. We propose that pre-conditioning of recipients using donor derived ECP treated cells prior to SOT plus the continued use of ECP performed on the recipient’s cells following transplant will provide sufficient protection under which IS medications can be confidently tapered and, in some instances, discontinued over time.

The presence and phenotype of DCs within the graft is known to be important in the development of rejection. Tolerogenic DCs induce T-cell anergy, can induce and expand regulatory T-cells, and induce apoptosis of memory T-cells. In this study, we observed an increase in graft-infiltrating DCs that express tolerogenic phenotype, e.g., significantly reduced CD86 in the ECP-DL treated allografts. An increase in CD86 expression has been shown to inhibit Tregs and co-stimulate T-cell responses, while CD80/CD152 engagement promotes tolerance. An initial increase of CD80 expression is consistent with observations that while reduction of CD86 expression favors tolerance, CD80 is necessary to engage with CTLA-4 to see enhanced inhibitory function of DCs. The subsequent decrease of CD80 expression over time may be related to the fluidity of the CD28/CTLA-4/co-stimulatory system^31^.

This is also the first time increased expression of MerTK on macrophages isolated from the grafts of ECP-treated mice have been shown. MerTK is expressed on macrophages and mediates binding of phagocytes to apoptotic bodies, triggering both anti-inflammatory and pro-repair cascades^32^. MerTK has also been shown to activate the phosphoinositol 3-kinase AKT pathway which down regulates NF-κB, a critical transcription factor in regulation of gene expression in inflammatory states. The presence of MerTK + myeloid derived suppressor cells in our system with down-regulated Ly6C and co-stimulatory molecules CD86, CD80, CCR2 (chemokine receptor on macrophages, mediates chemotactic response), and CXCR1 (chemokine receptor, mediates IL-8, a neutrophil chemotactic factor) suggests a polarization of myeloid cells from pro-inflammatory macrophages towards an anti-inflammatory reparative phenotype. These experiments are the first to demonstrate the induction of efferocytosis by ECP treatment. These findings are similar to those seen by our group in prior experiments utilizing donor cells rendered apoptotic by 1-ethyl-3-(-3dimethylaminopropyl) carbodiimide, or ECDI; MerTK expressed on macrophages was necessary to inhibit pro-inflammatory signaling and promoted expansion of myeloid derived suppressor cells and regulatory T-cells^33^.

Cellular therapy, utilized in the proper context and combination, represents the future of immune system hypo-responsiveness and perhaps even tolerance induction in the field of transplantation^3,34^. In our model, infusion of unmanipulated donor splenocytes did not protect against graft rejection; treatment with 8-MOP and UV-A light was required. Infusion of ECP-treated recipient cells prior to transplant also did not protect against rejection, indicating the effect is not simply related to the infusion of apoptotic cells. Other cellular therapy approaches utilizing either host or donor cells to induce tolerance are being investigated, including co-transplant with donor hematopoietic stem cells^4,5^, modified autologous Tregs^35–37^, ex-vivo culture of autologous or donor derived monocytes to create tolerogenic dendritic cells (“transplant acceptance-inducing cells”)^38–41^, and ex-vivo treatment of donor cells with Mitomycin C^6–8^. Many of these approaches have been studied in the The ONE Study, where a variety of cellular products were administered prior to living donor kidney transplant, including polyclonal regulatory cells, donor antigen reactive Tregs, tolerogenic DCs, and one using regulatory macrophage cells^11^. All products in this report, except for the regulatory macrophage, were of recipient origin. Although this study confirmed the feasibility and safety of the cell products used, larger scale studies will be needed to assess efficacy, the potential need for repeated cell infusions, the “best” cell type to use in different organ transplant situations, and the compatibility of concomitant IS agents with the cell product used.

We believe ECP to be superior to other methods currently being investigated for tolerance induction prior to transplant given its induction of immunologic cell death, long-term safety profile, potential for immediate clinical application, and ongoing treatment potential of the host after transplant without needing to re-approach the donor for additional cell infusions. The currently utilized ECP clinical procedure in the setting of SOT involves the treatment of the patient’s own peripheral white blood cells and could provide essential maintenance of immune system modulation over time after the transplant. The development of biomarkers in these patients could potentially guide this practice. Standard ECP treatment following transplant may further enhance the patient’s tolerogenic phenotype and maintain tolerance with the potential to reduce, or in some cases, eliminate additional immunosuppressive therapy, overall revolutionizing the approach to achieving transplant tolerance.

Several questions remain regarding optimization of this approach, providing rich potential for future research in the field. First, the concern regarding the potential of ECP-DL infusion to create sensitization that may promote antibody-mediated rejection. Patients undergoing ECP therapy for lung transplant demonstrate a reduction of DSA production over time^42^. In our hands, ECP-DL did not cause acute sensitization, rather, it significantly inhibited de novo DSA (dnDSA) production, likely due to its immunomodulatory effect on T-cells. dnDSA levels did rise over time without additional IS therapy but was significantly lower in the ECP-DL group. Further investigations are warranted to investigate whether dnDSA is responsible for late rejection and whether addition of post-transplant standard ECP or low dose IS therapy will prevent dnDSA production over time. A second question involves the evaluation of immune response kinetics, and timing of cell infusions. Precedent exists for cryopreserving autologous mononuclear cells with subsequent thawing and ECP-treatment^43–45^, so there can be opportunities to give subsequent doses of ECP-DL following transplant. Our data in rats receiving ECP-treated host cells post- liver transplant supports the approach of standard ECP after SOT and can be compared to re-treatment with donor cells. Next, we demonstrated that ECP-treated cells are processed primarily in the spleen, raising the question of whether the spleen is necessary for these interactions to occur. Finally, additional work evaluating infusion time points closer to the day of transplant could allow for a version of this protocol to be applied in the setting of deceased donor transplantation, particularly given the capacity to maintain grafts for 24 h following procurement. In summary, we believe the treatment of *donor* cells with ECP prior to SOT has significant promise to induce hypo-responsiveness of the recipient’s immune system, allowing judicious use and taper of IS. Thereafter, standard ECP treatment of recipient cells could serve to prolong the effect, potentially indefinitely.

## Materials and methods

### Animals

The Lewis (RT^1l^) rat breeders purchased from Charles River (Skokie, IL) and ACI (RT1^av1^) rat breeders from Harlan Sprague Dawley (Indianapolis, IN) and were bred in the specific-pathogen-free facility at the Center for Comparative Medicine of Northwestern University, and were used as recipients and donors, respectively at 10 to 14-week-old age. Ten to 14-week-old male BN (RT^1n^) rats were purchased from Charles River (Skokie, IL) and were used as a third-party control for this study. Ten to fourteen-week-old male BALB/c (H2^d^), B6 (H2^b^) and C3H (H2^k^) mice were purchased from the Jackson Laboratory (Bar Harbor, ME); C3H mice served as third party controls for this study. Thy1.1. 2C and 4C transgenic mice were provided by Dr. Xunrong Luo (Duke University). NOD.Cg-Prkdcscid Il2rgtm1Wjl/SzJ mice (8–14-week-old), also known as NSG mice, were purchased from the Jackson Laboratory. Animals were neither randomized nor blinded for experiments.

### Mouse heterotopic cardiac transplant & monitoring

Abdominal heart transplantation was performed as described previously^13^. In brief, the donor’s heart was excised en-bloc via median sternotomy. The ascending aorta and pulmonary artery of the donor were anastomosed end-to-side to the recipient abdominal aorta and inferior vena cava, respectively. Direct abdominal palpation of the heart was performed daily by two trained individuals. The graft survival time is defined as the post-operative day (POD) when complete loss of palpable cardiac impulses is observed.

### Tolerance induction by ECP treated donor-derived splenic leukocytes (ECP-DL)

ECP treatment involves four steps. Step 1, donor leukocytes are collected from the spleen of organ/tissue donors and processed with standard methods to make single cell suspension. Step 2, cells are incubated with 8-methoxtpsoralen at 200 ng/ml for 30 min. Step 3 treated cells are subject to UVA exposure of 4 J at room temperature in a closed light chamber (light chamber provided by Therakos, Inc, Mallinckrodt). Step 4, cells are rendered as ECP-DL through washes and re-suspensions and are infused intravenously into recipients at 1–7 days prior to transplantation. Cell doses administered prior to transplant were 5 X 10^7 in mouse (average weight 25 g) and 1 X 10^8 in rat (average weight 250 g) experiments. In an additional group of rats undergoing liver transplantation, peripheral blood leukocytes isolated from 200ul blood drawn from recipients at POD14 and POD21 underwent ECP treatment to become ECP-RL and re-infused on the same day.

### Immunosuppressive drug therapy

Rapamycin (RAPA; Sigma) solution was prepared by dissolving Rapamycin (RAPA) 1 mg in 4 mL of 0.2% Carboxyl Methylcellulose by sonication and given at 0.1 mg/kg/d, intraperitoneal to recipient mice from the day prior to transplant (d -1) to POD8. Subcutaneous tacrolimus (TAC, Astellas Pharma US, Inc.), 1 mg/kg daily, was administered from day -1 to POD8 (heart), or day -1 to POD8 (liver).

### Cell isolation and phenotypical analysis

Leukocytes were isolated from serially collected peripheral blood samples, spleens or liver grafts. Splenocytes were isolated using standard protocol. To obtain single cell-suspensions from cardiac or liver grafts, tissue was cut into small pieces (2 mm) and digested with collagenase IV (Worthington Biochemical Corporation, Lakewood, NJ). The resulting suspension was run through a 70 μm filter and washed with PBS. After centrifugation, the leukocytes were purified using lymphocyte separation medium (Fisher Scientific, Pittsburgh, PA). Phenotypical analysis was performed by using several panels of fluorescein-labeled anti-mouse mABs (against CD3, CD4, CD8, Ly6G, Ly6C, MHCII, CCR2, CXCR1, CD80, CD86) or rat monoclonal antibodies (CD3, CD4, or CD8). All antibodies were purchased from BD Biosciences. Frequencies of T-regulatory cells were identified using APC conjugated CD4, FITC conjugated-CD25, and PE conjugated-Foxp3 (Biolegend) monoclonal antibodies according to the manufacture instructions. Staining was performed with antibodies above (1 μg/10^6^ cells) at 4 °C for 30 min, washed, and analyzed by FACS (BD Biosciences).

### Adoptive transfer of transgenic T-cells

TCR transgenic 4C (Thy1.1 +) CD4^+^ T-cells or 2C CD8^+^ T-cells were purified from spleens of respective TCR transgenic mice using a CD4 or CD8 negative isolation kit (Miltenyi Biotec)^27,46^. 4C CD4^+^ T-cells or 2C CD8^+^ T-cells were labeled with 5 mM CFSE (Molecular Probes) and injected i.v. into CD45.1 + Thy1.2 + B6 congenic recipients on day -8 relative to the day of heart transplantation (day 0) and analyzed on indicated days (day-3 or day 5) following ECP-DL treatment.

### Graft histology and immunohistochemistry

All cardiac and liver grafts were harvested at the end of study for histological examination. Tissue samples were bisected transversely and placed in phosphate buffered 10% Formalin for 10–12 h. The tissues were embedded in melted paraffin using plastic cassettes. The sections were stained with H&E or PAS or Trichome Masson for morphologic evaluation. H&E-stained sections were examined under light microscopy by a blinded pathologist. Histopathological features of abnormal graft histology and acute rejection were assessed with established criteria^47^. Additional tissue was snap frozen in OCT compound with liquid nitrogen. All sections were 4 μm thick and blocked with either 10% donkey or goat serum (Sigma-Aldrich). Sections were stained with anti-mouse CD3, anti-mouseB220 or anti-mouse F4/80 mAbs (BD Biosciences), followed by goat anti-rat IgG Dylight 594 (1:500, Jackson ImmunoResearch) by NU’s mouse phenotyping core facility.

### Measurement of anti-donor antibody response

Anti-donor antibody levels were assessed as described previously^48^. Plasma samples were collected at days 7-, 14-, 21-, and 28-days post-transplantation. Splenocytes from Balb/c donors were incubated with plasma samples for one hour, and then stained with rat-anti-mouse IgG1, IgG2a/b, and IgG3. Addition of anti-mouse B220 antibody was used for gating out B cells. IgG levels were compared based on mean fluorescence intensity (MFI).

### Rat orthotropic liver transplant & post-transplant monitoring

Liver grafts from Lewis (isografts) or ACI rats (allografts) were transplanted orthotopically into Lewis recipient rats using a modified two-cuff technique as previously described^49^. Liver grafts were harvested and perfused thoroughly via the portal vein with cold heparinized (50U/ml) lactated Ringer's solution. The recipient’s native liver is removed and followed by suprahepatic vena cava anastomosis with a 10–0 running suture. The cuff technique was used for anastomosis of both the intrahepatic vena cava and portal vein. Common bile duct reconstruction was achieved by using a polyethylene stent. Recipients were monitored daily for clinical signs and symptoms of allograft rejection, including gradual weight loss, hunched posture, ruffled fur, reduced mobility, and jaundiced skin. Blood samples were collected periodically for multi-lineage phenotyping and liver function panel analysis (by Charles River Laboratories, Wilmington, MA). The day of transplantation was referred to as day 0; the endpoint of study was defined as > 180 post-operative days of survival, recipient death or when clinical signs of liver failure from rejection developed.

### Rat orthotopic kidney transplant & post-transplant monitoring

Kidney grafts from Lewis (isografts) or ACI rats (allografts) were transplanted orthotopically into bi-nephrectomized Lewis recipient rats; using the technique as previously described^29,30^. In brief, one native kidney was removed on the day of transplant; donor aorta and inferior vena cava were anastomosed to recipient aorta below the level of the native renal vessels. The bladder patch from the donor is anastomosed to the dome of the recipient’s bladder. The second native kidney was removed on post-transplant day + 4. Given the recipient rats are ultimately bi-nephrectomized, their survival depends on the function of the transplanted kidney. Recipients were monitored daily for clinical signs and symptoms of allograft rejection, including gradual weight loss, hunched posture, ruffled fur, reduced mobility, and jaundiced skin. Blood samples were collected periodically for multi-lineage phenotyping and kidney function panel analysis (by Charles River Laboratories, Wilmington, MA). The day of transplantation was referred to as day 0; the endpoint of study was defined as > 180 post-operative days of survival, recipient death or when clinical signs of kidney failure from rejection developed.

### Statistical analysis

All analyses were done with GraphPad PRISM 7 software (https://www.graphpad.com/scientific-software/prism/). Comparisons between graft survival times were calculated using Kaplan–Meier survival curves with the log-rank test. Flow cytometry analyses are expressed as the mean ± standard deviation (SD). Statistical significances between the groups were determined by Wilcoxon nonparametric tests or by an unpaired Student’s t-test with significance determined at P < 0.05 (***P =  ≤ 0.001; **P =  < 0.01; *P =  < 0.05). The data representing more than 2 groups were analyzed with one-way ANOVA analysis.

### Study approval

Animals were housed under specific pathogen-free conditions. All surgical procedures and research protocols were approved by the institutional animal care and use committee (IACUC) of Northwestern University, and in accordance with ARRIVE guidelines.

## Supplementary Information

Supplementary Figure Legends.

Supplementary Figures.
