# Supplementary material for: Pre-transplant infusion of donor leukocytes treated with extracorporeal photochemotherapy induces immune hypo-responsiveness and long-term allograft survival in murine models
Source: Sci Rep. 2022 May 4;12:7298. doi: 10.1038/s41598-022-11290-w (PMC9068706; doi:10.1038/s41598-022-11290-w)
Supplement: Supplementary file 1 — Supplementary Figure Legends. [file 41598_2022_11290_MOESM1_ESM.docx]

**Supplemental Fig 1.** (**a**) Kaplan Meier Curves showing similar protection achieved with ECP-DL infusion at days -3 or -1 as those given on day -7 (n=4-11/group) in the heterotopic heart transplant model. (**b**) Kaplan Meier Curves illustrating that the protective effect of ECP-DL was independent of strain combination, as the reverse donor to recipient pair (B6 to BALB/c) had prolonged graft survival like those noted in the primary experiments (n=5/group).

**Supplemental Fig 2. A single ECP-DL infusion decreases antigen-specific CD8^+^ T-cells and suppresses their proliferation following cardiac transplantation.** (**a**) Experiments described in Figure 3 were reproduced in subsequent animal groups, in which the B6 mice receiving 2C CD8 T-cell transfer and ECP treated/untreated DL infusion went on to heterotopic cardiac transplantation. Animals were sacrificed at post-operative day 5 and CD8 T-cells were assessed within the graft and spleen. (**b**) Representative flow cytometric plots demonstrating the heart graft and spleen retrieved from recipient mice treated with ECP-DL had substantially lower numbers of antigen-specific CD8 cells (2C+) compared to the untreated group.

**Supplemental Fig 3. ECP-treated cells became apoptotic prior to infusion and were phagocytosed in the spleen.** (**a**) Approximately half of the ECP-treated spleen cells, including antigen-presenting cells (APCs; gated from MHCII+ cells) and T-cells (gated from CD3+ cells), were apoptotic at the time of infusion, as determined by Annexin V staining. (**b, c**) To determine the fate of ECP-DL in vivo, cells were labeled with PKH26 dye and injected into B6 mice (n=4). Spleens were harvested 15 hours after ECP-DL infusion. PKH26 signal intensity was tested in CD45+ leukocytes and subpopulations, including monocytes (CD11b+Ly6C+), granulocytes (CD11b+Ly6G+) and macrophages (CD11b+F4/80+). PKH26 positive cells accounted for around 6% of total splenic leukocytes. Within the myeloid cell populations, the PKH26 signal was detected mainly in macrophages but not in granulocytes or monocytes. These results indicate that ECP-treated cells rendered apoptotic were at least in part phagocytosed by macrophages in the spleen.

**Supplemental Fig 4.** Gating strategy for cellular infiltrates isolated from cardiac grafts to perform quantitative and phenotypic experiments described in Figs 5, 6, and Supplemental Fig 5.

**Supplemental Fig 5. Pre-treatment with ECP-DL led to reduced expression of co-stimulatory molecules CD80 and CD86, suggesting a polarization of graft-infiltrating myeloid cells from pro-inflammatory to anti-inflammatory phenotypes.** BALB/c to B6 cardiac allografts untreated or treated with ECP-DL were sacrificed on POD6 or 12, and graft-infiltrating cells were subjected to flow cytometry analysis. Cells are gated on H-2b+ (host) CD11b+ live singlets (n=3/group). (**a-c**) Representative dot-plots and corresponding bar graphs showing reduced graft-infiltrating myeloid cells, Ly6G+Ly6Cint (granulocytes) and Ly6G-Ly6Chi cells (monocyte/macrophages), in the ECP-DL treated group at POD6. (**d-k**) Representative histogram and corresponding bar graphs demonstrating the expression of costimulatory molecules (CD80 and CD86) and MHC class II (I-Ab) in both granulocytes (**d-g**) and monocyte/macrophages (Mono/Macs) (**h-k**) at POD6 and POD12. MFI: Mean fluorescence intensity; *p<0.05, **p<0.01, ***p<0.001 by student *t*-test.

**Supplemental Fig 6. ECP-DL infusion 7 days prior to MHC-mismatched orthotopic kidney transplant resulted in prolonged graft survival with kidney function comparable to immunosuppression alone, with histologic examination comparable to isograft controls.** Native kidneys were removed from recipient Lewis rats; a single kidney from donor ACI rats was transplanted. Recipients were untreated (n=8) or treated with ECP-DL alone (n=7), TAC (n=6), ECP-UL (n=3), or ECP-DL plus TAC (n=6). (**a**) Kaplan Meier Curves showing that animals receiving ECP-DLs alone had superior survival to those treated with TAC alone. Those receiving ECP-DLs plus TAC survived indefinitely, illustrating a synergistic effect. ECP-DL versus Untreated, ****p<0.0001; ECP-DL versus ECP-UL, **p<0.01; ECP-DL/TAC versus ECP-DL, p=0.08, ECP-DL/TAC versus TAC, *p<0.05 by Log-rank test. (**b**) The creatinine levels indicating ECP-DL improved renal allograft function. ***p<0.001, ****p<0.0001 versus Untreated group, by Student *t*-test. (**c**) Histology at the time of sacrifice demonstrated an extensive cellular infiltrate and obliteration of normal kidney architecture in the untreated animals, whereas kidney tissue from the ECP-DL group appeared like the isograft group at POD200.
