# Supplementary material for: Pre-transplant infusion of donor leukocytes treated with extracorporeal photochemotherapy induces immune hypo-responsiveness and long-term allograft survival in murine models
Source: Sci Rep. 2022 May 4;12:7298. doi: 10.1038/s41598-022-11290-w (PMC9068706; doi:10.1038/s41598-022-11290-w)

Supplemental figure 1

a

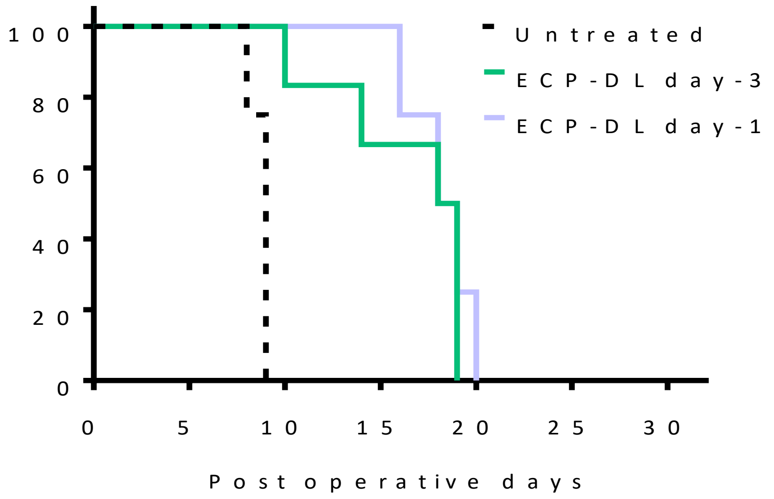

b

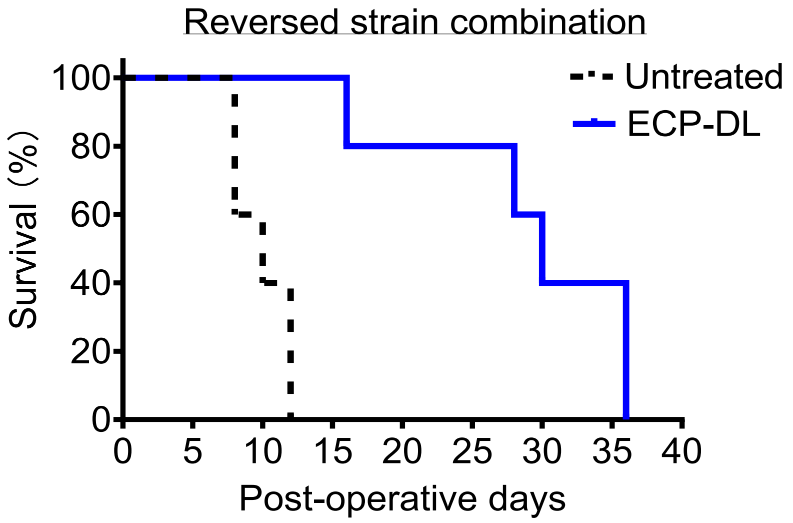

Supplemental figure 2

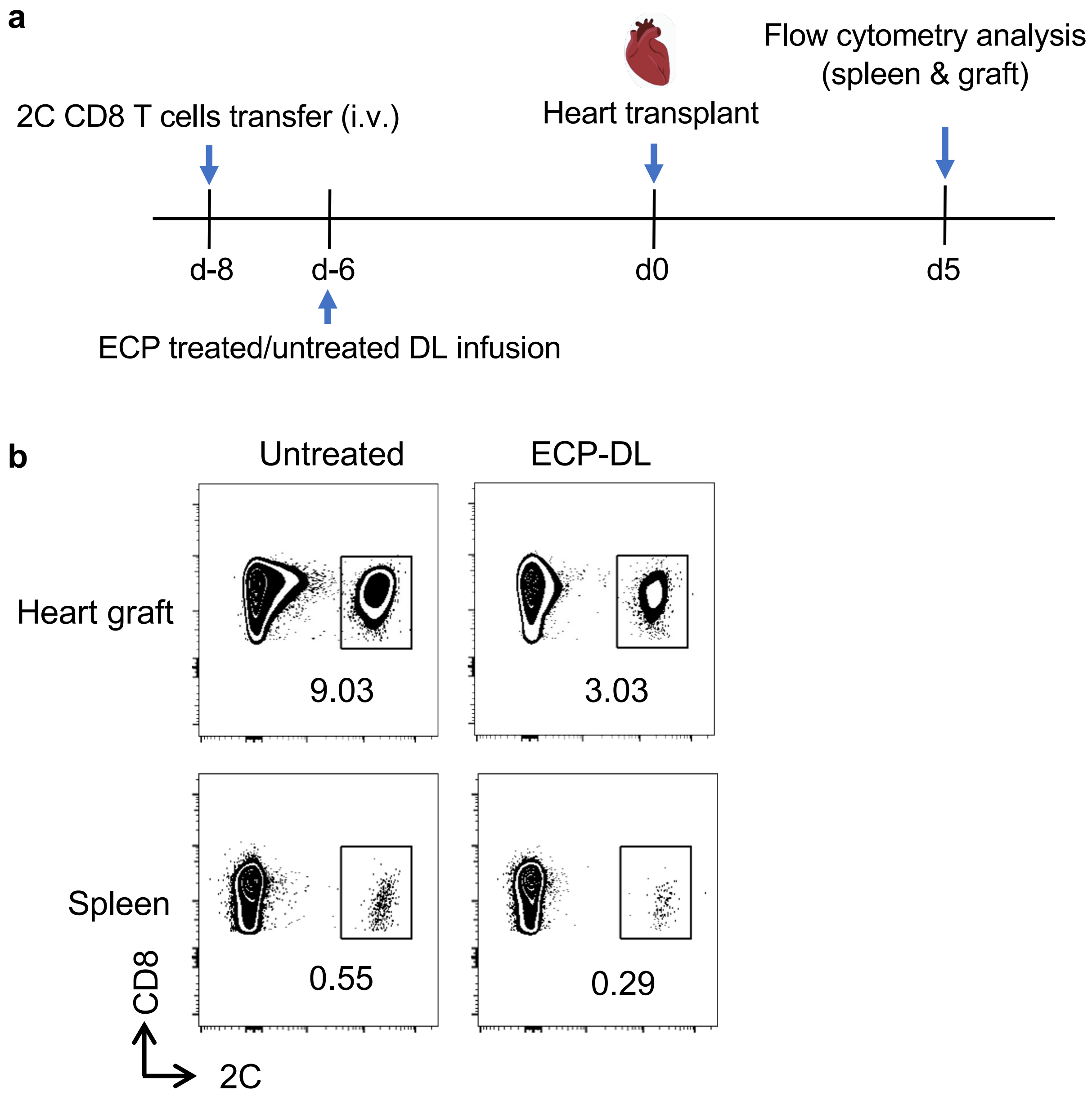

Supplemental figure 3

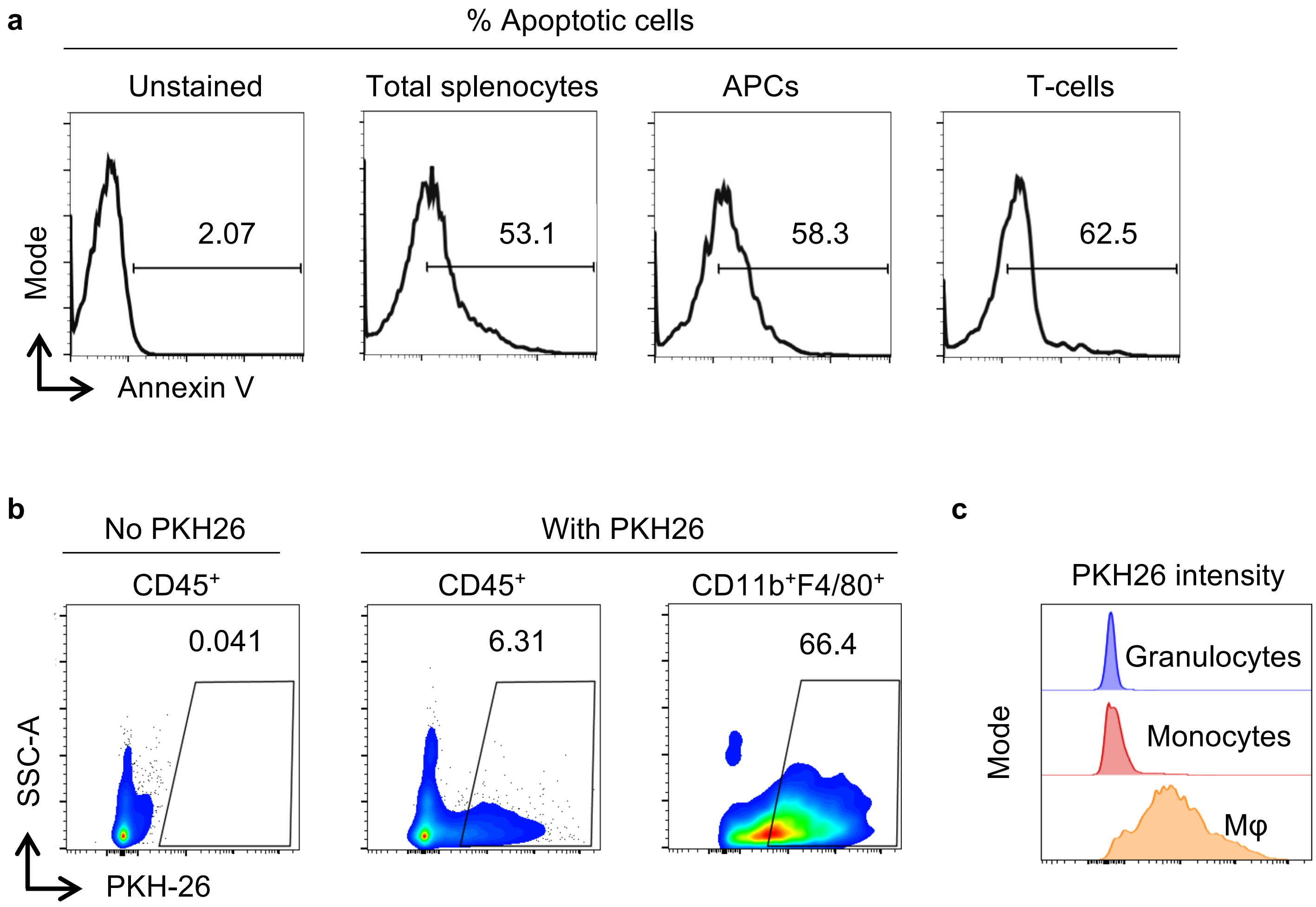

Supplemental figure 4

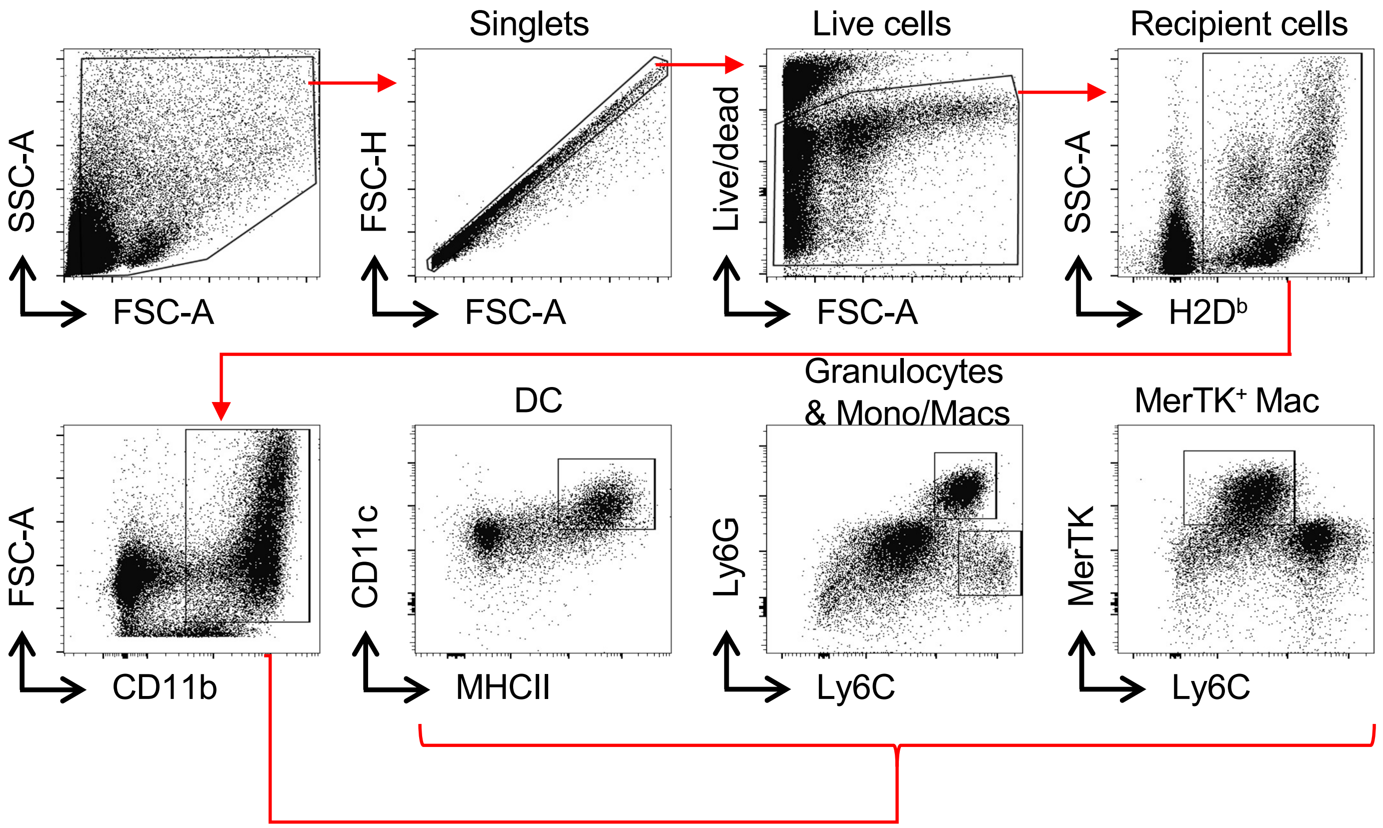

Supplemental figure 5

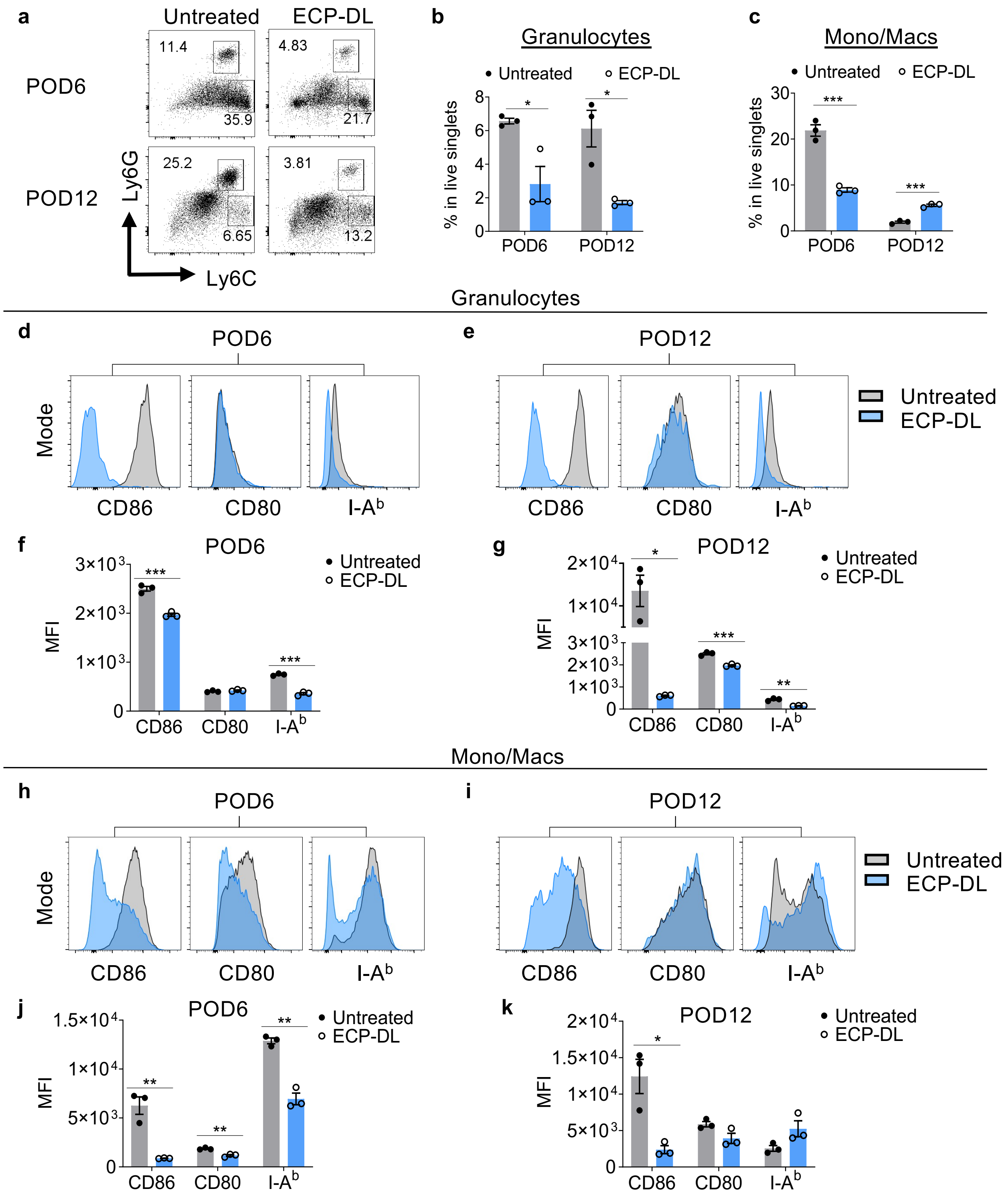

Supplemental figure 6

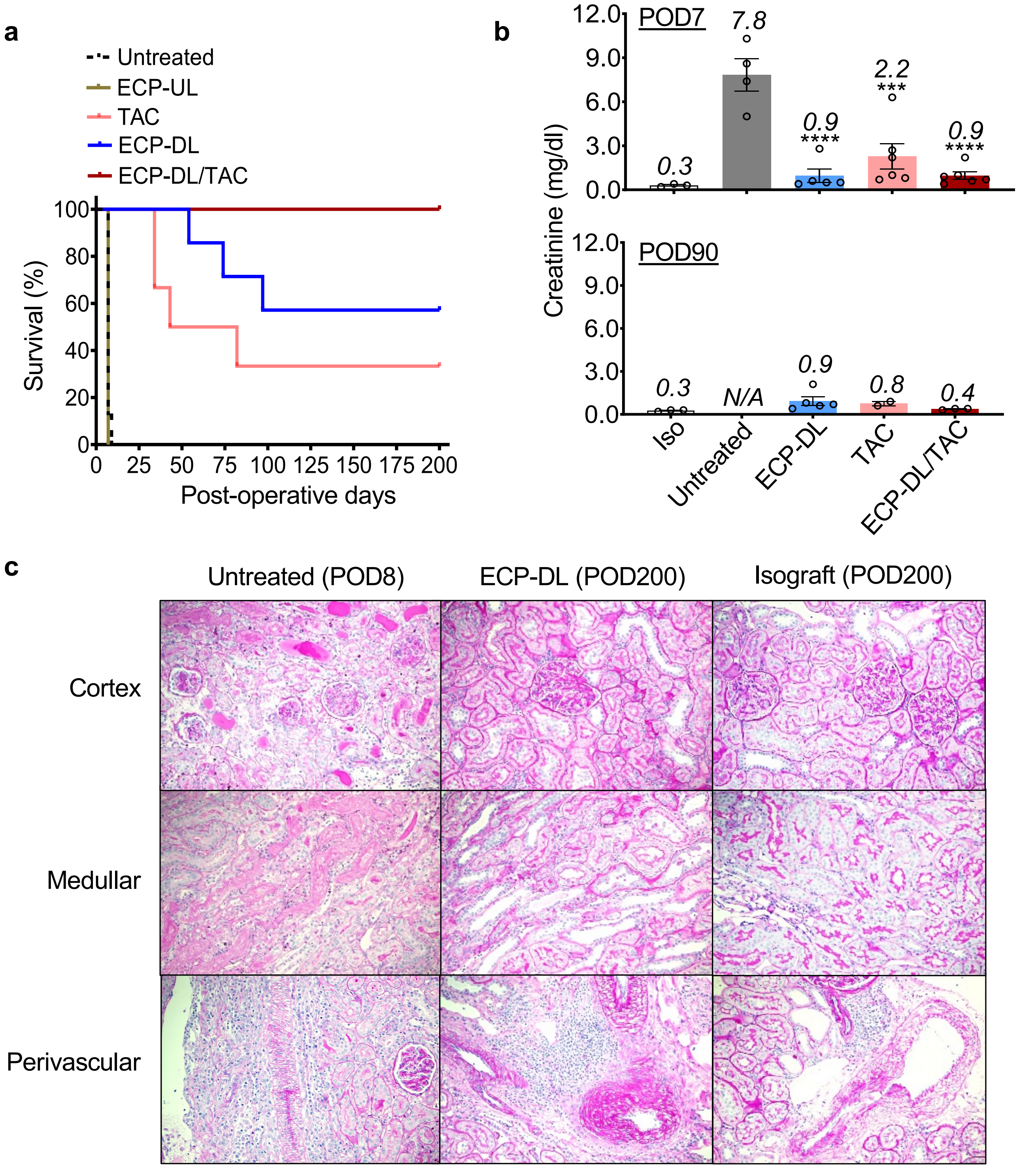

Supplement: Supplementary file 2 — Supplementary Figures. [file 41598_2022_11290_MOESM2_ESM.pdf]
